# Collagen XIX Alpha 1 Improves Prognosis in Amyotrophic Lateral Sclerosis

**DOI:** 10.14336/AD.2018.0917

**Published:** 2019-04-01

**Authors:** Ana C. Calvo, Gabriela Atencia Cibreiro, Paz Torre Merino, Juan F. Roy, Adrián Galiana, Alexandra Juárez Rufián, Juan M. Cano, Miguel A. Martín, Laura Moreno, Pilar Larrodé, Pilar Cordero Vázquez, Lucía Galán, Jesús Mora, José L. Muñoz-Blanco, María J. Muñoz, Pilar Zaragoza, Elena Pegoraro, Gianni Sorarù, Marina Mora, Christian Lunetta, Silvana Penco, Claudia Tarlarini, Jesús Esteban, Rosario Osta, Alberto García Redondo

**Affiliations:** ^1^LAGENBIO (Laboratory of Genetics and Biochemistry), Faculty of Veterinary-IIS, IA2-CITA, University of Zaragoza, Zaragoza, Spain.; ^2^Neurology Department, ALS Unit, CIBERER U-723, Health Research Institute, October 12th Hospital “IIS I+12”, Madrid, Spain.; ^3^Ferkauf Graduate School of Psychology, Yeshiva University, NY 10461, USA.; ^4^Servicio de Reumatología, Hospital Universitario de la Princesa, Instituto de Investigación Sanitaria La Princesa, Madrid, Spain.; ^5^Orthopaedic Surgery Department, October 12th Hospital, Madrid, Spain.; ^6^Grupo Enfermedades Mitocondriales y Neuromusculares, Instituto de Investigación Sanitaria Hospital 12 de Octubre (imas12), U723-CIBERER, Madrid, España.; ^7^Neurology Department, ALS Unit, Clínico Universitario San Carlos Hospital, Madrid, Spain.; ^8^Neurology Department, ALS Unit, Carlos III Hospital, Madrid, Spain.; ^9^Neurology Department, ALS Unit, Health Research Institute, Gregorio Marañón Hospital “IISGM”, Madrid, Spain.; ^10^Neurological Clinic, Department of Neurosciences, University of Padova, Padova, Italy.; ^11^Muscle Cell Biology Laboratory, Neuromuscular Diseases and Neuroimmunology Unit, Fondazione IRCCS Istituto Neurologico C. Besta, Milan, Italy.; ^12^NEMO (NEuroMuscular Omnicentre) Clinical Center, Fondazione Serena Onlus, Milan, Italy.; ^13^Medical Genetics Unit, Department of Laboratory Medicine, Niguarda Ca’ Granda Hospital, Milan, Italy.

**Keywords:** Collagen XIX type A1, ALS, prognostic biomarker, neurodegeneration, disease progression

## Abstract

The identification of more reliable diagnostic or prognostic biomarkers in age-related neurodegenerative diseases, such as Amyotrophic Lateral Sclerosis (ALS), is urgently needed. The objective in this study was to identify more reliable prognostic biomarkers of ALS mirroring neurodegeneration that could be of help in clinical trials. A total of 268 participants from three cohorts were included in this study. The muscle and blood cohorts were analyzed in two cross-sectional studies, while the serial blood cohort was analyzed in a longitudinal study at 6-monthly intervals. Fifteen target genes and fourteen proteins involved in muscle physiology and differentiation, metabolic processes and neuromuscular junction dismantlement were studied in the three cohorts. In the muscle biopsy cohort, the risk for a higher mortality in an ALS patient that showed high Collagen type XIX, alpha 1 (COL19A1) protein levels and a fast progression of the disease was 70.5% (*P* < 0.05), while in the blood cohort, this risk was 20% (*P* < 0.01). In the serial blood cohort, the linear mixed model analysis showed a significant association between increasing *COL19A1* gene levels along disease progression and a faster progression during the follow-up period of 24 months (*P* < 0.05). Additionally, higher *COL19A1* levels and a faster progression increased 17.9% the mortality risk (*P* < 0.01). We provide new evidence that *COL19A1* can be considered a prognostic biomarker that could help the selection of homogeneous groups of patients for upcoming clinical trial and may be pointed out as a promising therapeutic target in ALS.

Amyotrophic Lateral Sclerosis (ALS) is a devastating neurodegenerative disease that promotes a progressive motor neuron loss and muscle weakness. The survival period of time is within 2 to 10 years after the signs and symptoms firstly appear [1,2]. A large proportion of people with ALS are sporadic cases and they lack of clear genetic association, while a small proportion of ALS patients, 5-10%, correspond to familial ALS cases and they have a family history of ALS or a related condition called frontotemporal dementia (FTD). FTD is a progressive brain disorder that affects personality, behavior, and language and this disorder is present in 20% of the ALS patients [1]. The most known mutations that produce the typical adult-onset ALS phenotype in the familial cases are related to the copper/zinc superoxide-dismutase-1 gene (*SOD1* ), Tar DNA-binding protein gene (*TARDBP* ) (previously known as TDP-43), DNA/RNA-binding protein called *FUS* (fused in sarcoma), *TLS* (translocation in liposarcoma), and the most recent hexanucleotide repeat expansion in *C9ORF72* [1,3].

The complexity in the discovery of potential biomarkers for neurodegenerative diseases, especially in the case of ALS, relies greatly upon the lack of etiopathogenic origin in the whole population of ALS patients. In fact, ALS shares physiophatological abnormalities with other neuropathies such as Alzheimer’s disease or Parkinson’s disease and, more precisely, this close connection among these neurodegenerative diseases makes the identification of specific biomarkers for ALS a difficult task [4,5]. Dementia is also relatively frequent in ALS and may be a consequence of either frontotemporal lobar degeneration (FTLD) or a result of co-existing Alzheimer disease [6,7].

In this complex scenario and in spite of the numerous studies attempting to find specific gene/protein targets exclusive for ALS and characteristic of both familial and sporadic cases, the prognosis of the disease remains poor. Another main challenge relies on the fact that ALS is a multifactorial and relentlessly progressive disease, which hinders the use of an effective treatment. Therefore, the search for reliable biomarkers of the disease that can provide the identification of accurate indicators of early symptoms, disease progression, or even patients’ survival, is urgently needed. It has been reported that older age at symptom onset, early respiratory muscle dysfunction, and bulbar-onset disease are associated with reduced survival, whereas limb-onset disease, younger age at presentation, and longer diagnostic delay are independent predictors of prolonged survival [8]. In this study, our main objective was to identify molecular biomarkers mirroring neurodegeneration in ALS patients that can also enable an earlier prognosis in the disease.

Previous studies by our research workgroup on transgenic SOD1G93A mice suggested five genes, *Mef2c* (myocyte enhancer factor 2C), *Gsr* (oxidative stress metabolism), *Col19a1* (collagen, type XIX, alpha 1), *Calm1* (calmodulin 1), and *Snx10* (sorting nexin 10), as potential genetic biomarkers of longevity in transgenic SOD1G93A mice, one of the best-characterized animal models for ALS that resembles both clinical and pathological characteristics of ALS patients [9,10]. Consequently, our next and main challenge was to analyze in depth the prognostic nature of the biomarkers identified in this animal model in ALS patients to correlate these biomarkers to the clinical variables routinely monitored in ALS patients to improve accuracy in the prediction. *Mef2c* gene is involved in muscle differentiation and regeneration [11], *Gsr* and *Calm1* genes are involved in oxidative stress [12] and calcium homeostasis [13], respectively, *Col19a1* gene is involved in the maintenance of muscle integrity [14] and *Snx10* gene can play a relevant role in the muscle and bone dysregulation [15]. To achieve this challenge, we firstly aimed to test a panel of biomarkers in muscle biopsies from ALS patients and secondly, in blood samples to identify broadly applicable prognostic biomarkers for ALS disease progression in a non-invasive way. More in depth, the specific objective in this study was to look at the associations among molecular markers and clinical variables to identify more reliable prognostic biomarkers that could be of help in clinical trials. The findings in this study revealed that *COL19A1* levels in muscle biopsies and blood samples from ALS patients can be considered a prognostic biomarker to accurately monitor the disease progression, especially in those patients that share a high disability in the first symptomatic stages of the disease.

## MATERIALS AND METHODS

### Study approval

Blood and muscle biopsy samples from patients were obtained with written informed consent prior to inclusion in the study to publication of their case details, which has been conducted according to Declaration of Helsinki principles, following the ethical rule of the October 12th Hospital (local Ethical Committee of Clinical Investigation approval reference 14/2007, Madrid, Spain), and according to the Directive 2004/23/EC of the European Parliament and of the Council. Participants were identified by number, not by name.

### Experimental Design

**Figure 1.**
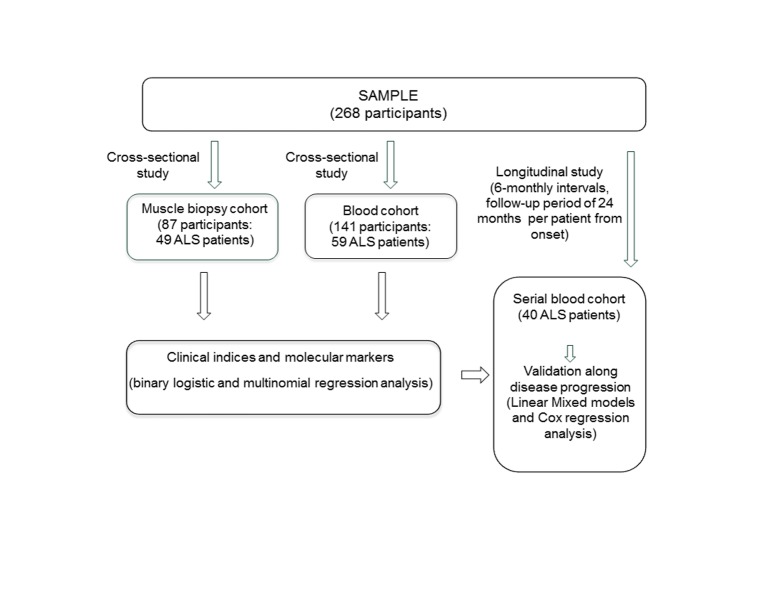
Flow diagram of the three study cohorts and overview of the study design. A total of 148 ALS patients were enrolled in two cross-sectional studies and one longitudinal study to detect associations among molecular markers and clinical variables in skeletal muscle biopsy (ALS muscle biopsy cohort) and blood samples (ALS blood cohort) and to identify prognostic biomarkers along the disease progression, especially in serial blood samples.

This study included a total of 268 participants from three cohorts, matched for age and gender, whenever possible. For the muscle biopsy cohort, a total of 49 ALS patients, 24 healthy controls and 14 patients with other neuropathies (ONP) were included (Table 1). In the blood cohort, 59 ALS patients, 58 healthy controls and 24 ONP patients were included (Table 2). A second subgroup of forty sporadic ALS patients was monitored in the serial blood cohort at 6-monthly intervals during a follow-up period of 24 months from the symptom onset in each patient (Table 3). The muscle and blood cohorts were analyzed in two cross-sectional studies, while the serial blood cohort was analyzed in a longitudinal study. The participants were different in the three cohorts (Fig. 1).

**Table 1:** General and clinical characteristics of the study subjects in the muscle biopsy study cohort.

Type of cohort (N)		Muscle biopsy cohort* (N = 87)		
		ALS patients	ONP patients	Healthy controls
Patients' characteristics		(n =49)	(n = 14)	(n = 24)
Gender (n)	Female	15	8	19
	Male	34	6	5
Age at illness onset (mean ± SD)		57.02 ± 12.42	43.79 ± 16.02	
Age at biopsy (mean ± SD)		58.65 ± 12.52	42.34 ± 25.89	55.07 ± 26.07
Disease duration, months (mean ± SD)		19.54 ± 25.15		
Clinical Phenotype (n)	ALS, spinal	42		
	ALS, bulbar	6		
	ALS + FTD	1		
	OMD		5	
	SMA		5	
	CIDP		1	
	KD		1	
	NMD		1	
	AD (Early)		1	
ALSFRS-r at biopsy (mean ± SD)		40.24 ± 6.20		
*El Escorial* criteria at onset (n)	Unavailable	33		
	Defined	1		
	Probable	6		
	Possible	5		
	Suspected	4		
Genetic Diagnosis (n)	SALS	26		
	FALS	6		
	SALS + FTD	2		
	MND-FTD	1		
	PLS-SALS	1		
	PMA-SALS	1		
	Unavailable	12		

* All subjects were of caucasian ethic group.

ALS, amyotrophic lateral sclerosis; SALS, sporadic ALS; FALS, familial ALS; ALSFRS-r, the revised ALS Functional Rating Scale; ONP, other neuropathies; FTD, frontal temporal dementia; OPMD, oculopharyngeal muscular dystrophy; SMA, spinal muscular atrophy; CIDP, chronic inflammatory demyelinating polyneuropathy; KD, Kennedy Disease; MND, motor neuron disease (cervical myelopathy); AD, Alzheimer Disease (early); PLS, Primary Lateral Sclerosis; PMA, Primary Muscular Atrophy.

The clinical characteristics of the participants in each cohort were firstly assessed. In the ALS group, these characteristics included the revised ALS Functional Rating Scale-revised (ALSFRS-r), the ALSFRS-r slope (48-ALSFRS-r /disease duration at time assessment), the clinical phenotype, the diagnostic delay, the age at onset, age at sampling, gender, the revised El Escorial criteria [16], the onset site and the genetic diagnosis. The ALSFRS-r provided an estimation of the patient’s degree of functional impairment. In the serial blood cohort, ALSFRS-r was evaluated serially at 6-monthly intervals to objectively assess any progression of the disease at each sample extraction time. Additionally, the ALSFRS-r slope was calculated in both cross-sectional and longitudinal studies to monitor the rate of progression based on the functional scale of the patient. The classification system to estimate the ALSFRS-r slope was based on the previous system used by Elamin and coworkers [17]. The interpretation of this classification system is as follows: ALSFRS-R slope <0.025 points/month, 0.25-0.49 points/month, 0.50-0.99 points/month, and ≥1 points/month. This means the higher ALSFRS-r slope (≥1 points/month), the faster functional decline.

**Table 2:** General and clinical characteristics of the study subjects in the blood study cohort.

Type of cohort (N)		Blood cohort* (N = 141)		
		ALS patients	ONP patients	Healthy controls
Patients’ characteristics		(n = 59)	(n= 24)	(n = 58)
Gender (n)	Female	25	10	31
	Male	34	14	27
Age at illness onset (mean ± SD)		60.98 ± 14.07	43.79 ± 16.02	
Age at biopsy (mean ± SD)		62.10 ± 14.00	55.74 ± 12.32	55.74 ± 12.31
Disease duration, months (mean ± SD)		14.53 ± 14.66		
Clinical Phenotype (n)	ALS	59		
	CIDP		1	
	KD		1	
	NMD		1	
	AD (Early)		1	
	BMD		2	
	ES		1	
	FMD		4	
	HPP (Type-1)		1	
	LMD		1	
	MM		1	
	MD (Type-1)		9	
	MD (Type-2)		1	
Site at onset (n)	Lower limb	27		
	Upper limb	17		
	Bulbar	10		
	Generalized	4		
	Respiratory	1		
	None			
ALSFRS-r at onset (mean ± SD)		42.71 ± 6.86		
ALSFRS-r at biopsy (mean ± SD)		34.47 ± 7.82		
*El Escorial* criteria at onset (n)	Unavailable	18		
	Defined	17		
	Probable	18		
	Possible	3		
	Suspected	2		
Genetic Diagnosis (n)	SALS	33		
	FALS (4m**)	18		
	FALS (SOD1)	6		
	FALS (SETX)	1		
	Unavailable	1		

* All subjects were of caucasian ethic group except two latin-american and one asiatic.

** Four mutations for ALS are present: SOD1/TDP43/FUS/c9orf72. ALS, amyotrophic lateral sclerosis; ONP, other neuropathies; CIDP, chronic inflammatory demyelinating polyneuropathy; KD, Kennedy Disease; MND, motor neuron disease (cervical myelopathy); AD, Alzheimer Disease (early); BMD, Becker's Muscular Dystrophy; ES, extrapyramidal syndrome; FMD, Facio-capulo-humeral muscular distrophy; HPP, Hypokaliemic Periodic Paralysis; LMD, limb-girdle muscular distrophy; MM, mild myotonia; MD, Myotonic Dystrophy.

### RNA extraction and purification from muscle biopsies and blood samples

Muscle biopsy samples were mainly obtained from brachial biceps using open biopsy after subcutaneous anesthesia administration and the samples were immediately frozen in liquid nitrogen. The biopsy sample from each individual was used to obtain RNA and protein extracts, using used the same methodology as the one employed in a previous study [18].

Blood samples were immediately processed and treated with a Ficoll gradient (Ficoll-PaqueTM Plus; GE Healthcare, Madrid, Spain). RNA extraction and purification were carried out following the manufacturer’s instructions. In the case of blood samples received frozen in Pax tubes, the PAXgene Blood RNA Kit (PreAnalytiX, 8634 Hombrechtikon, Switzerland) was used to extract the RNA fraction. In all the muscle and blood samples, the cDNA was also obtained from 1 μg of total extracted RNA (High Capacity cDNA RT kit; Applied Biosystems, Madrid, Spain). One muscle biopsy sample and one blood sample were obtained per participant from the muscle biopsy and blood cohorts, respectively. In the serial blood cohort, serial blood samples obtained every six months. In this case, a maximum of five serial samples were collected per patient, starting at the first age of sampling.

### Gene expression analysis

The gene expression analysis was performed by real-time PCR (7500 Real-Time PCR System, Applied Biosystem, Madrid, Spain). Fifteen target genes were included in this analysis (Table 4). The Taqman probes (Applied Biosystems, Madrid, Spain) (Table 4) used in this study were tested and in all the cases the efficiency was near 100%. All the real-time PCR reactions were done in triplicate and three endogenous genes (glyceraldehyde 3-phosphate dehydrogenase, GAPDH, hypoxanthine phosphoribosyltransferase, HPRT, and TATA-binding protein, TBP) were used to normalize the target gene expression. Data was quantitatively analyzed with respect to a calibrator sample, selected from a control subject, using the ΔΔCt method.

**Table 3:** General and clinical characteristics of sporadic ALS patients in the serial blood study cohort.

Type of cohort (N)		Serial blood cohort * (N = 40)				
		ALS patients (n =40)				
Patients' characteristics		Onset	6 months	12 months	18 months	24 months
Gender (n)	Female	15				
	Male	25				
Age at illness onset (mean ± SD)		57.02 ± 12.42				
Disease duration, months (mean ± SD)		57.53 ± 23.48				
Clinical Phenotype (n)	SALS, UMN and LMN spinal	25				
	SALS, UMN spinal	1				
	SALS, LMN spinal	14				
ALSFRS-r (mean ± SD)		27.8 ± 5.47	23.8 ± 5.94	19.6 ± 6.27	18.5 ± 6.76	17.6 ± 6.76
*El Escorial* criteria at onset (n)	Defined	25				
	Possible	4				
	Probable	11				
Forced vital capacity (FVC) (%)		10.4 ± 6.11	14.6 ± 6.99	15.0 ± 10.44	17.8 ± 11.12	20.6 ± 15.74
Body mass index (BMI) (Kg/m^2^)		26.5 ± 3.81	26.3 ± 3.82	25.9 ± 4.11	26.0 ± 3.71	25.3 ± 3.55
Genetic Diagnosis (n)	SALS	40				

* All subjects were of caucasian ethic group. SALS, sporadic ALS; UMN, upper motor neuron; LMN, lower motor neuron; FVC, forced vital capacity; BMI, body mass index.

### Protein expression analysis

Fourteen target proteins were included in this analysis (Table 5). We used the same methodology as the one employed in a previous study [18]. In this study, the protein extracts were subjected to 10% SDS-polyacrylamide gel electrophoresis. The corresponding primary antibody was diluted 1:500 in blocking buffer and glyceraldehyde-3-phosphate dehydrogenase (GAPDH, Santa Cruz Biotechnology, Quimigen S.L., Madrid, Spain) was used as housekeeping protein to normalize protein levels in target proteins. The integrated optical density (IOD) values of the target proteins were accurately normalized with respect to the housekeeping protein IOD values.

**Table 4:** Taqman® probe and primer mixtures used in gene expression assays.

Gene name	Gene Symbol	Assay ID
Ankyrin repeat domain 1 (cardiac muscle)	*ANKRD1*	Hs00173317_m1
Collagen, type XIX, alpha 1	*COL19A1*	Hs00156940_m1
F-box protein 32	*FBXO32*	Hs01041408_m1
Glycogen synthase kinase 3	*GSK3*	Hs01047719_m1
Glutathione reductase	*GSR*	Hs00167317_m1
Inositol(myo)-1(or 4)-monophosphatase 1	*IMPA1*	Hs04188597_m1
Myocyte enhancer factor 2C	*MEF2C*	Hs00231149_m1
Myogenin (myogenic factor 4)	*MYOG*	Hs01072232_m1
Reticulon 4	*RTN4 (NOGO A)*	Hs00199671_m1
Ras-related associated with diabetes	*RRAD*	Hs00188163_m1
Senataxin	*SETX*	Hs00209294_m1
Sarcolipin	*SLN*	Hs01888464_s1
Sorting nexin 10	*SNX10*	Hs00203362_m1
Superoxide dismutase 1, soluble	*SOD1*	Hs00533490_m1
Vacuolar protein sorting 54 homolog (*S. cerevisiae* )	*VPS54*	Hs00212957_m1

**Table 5:** Primary antibodies used in protein expression assays.

Protein name	Protein Symbol	Reference number, primary antibodies
Anti-Apoptosis-inducing factor, mitochondrion-associated, 1	AIFM1	SAB2100079, anti-rabbit, Sigma-Aldrich
Ankyrin repeat domain 1 (cardiac muscle)	ANKRD1	SAB1101413, anti-rabbit antibody, Sigma-Aldrich
Aspartate transcarbamylase	CAD	SAB2100334, anti-rabbit, Sigma-Aldrich
Collagen, type XIX, alpha 1	COL19A1	H00001310-A01, anti-mouse, Novus Biologicals
Coenzyme Q3 homolog	CoQ3	WH0051805M1, anti-mouse, Sigma-Aldrich
F-box protein 32	FBXO32	ab67866, anti-mouse, ABCAM
Glutathione reductase	GSR	WH0002936M1, anti-mouse, Sigma-Aldrich
Inositol(myo)-1(or 4)-monophosphatase 1	IMPA1	SAB2103588, anti-rabbit, Sigma-Aldrich
Myogenin (myogenic factor 4)	MYOG	WH0004656M1, anti-mouse, Sigma-Aldrich
Anti-CoQ1	PDSS1	AV46195, anti-rabbit, Sigma-Aldrich
Reticulon 4	RTN4 (NOGO A)	R3282, anti-rabbit, Sigma-Aldrich
Sarcolipin	SLN	ab25860, anti-rabbit, ABCAMK
Sorting nexin 10	SNX10	WH0029887M1, anti-mouse, Sigma-Aldrich
Vacuolar protein sorting 54 homolog (S. cerevisiae)	VPS54	SAB1401701, anti-mouse, Sigma-Aldrich

### Statistical analysis

The normality of data was verified by means of the Kolmogorov-Smirnov test. The data that did not show a homogeneous distribution was analyzed using Kruskal-Wallis tests to compare means, while the homogeneous distribution of the data was studied using Student's-t test. All values were expressed as the mean ± standard error of the mean (SEM). The area under ROC curves (AUC) was calculated, following the same methodology used in a previous study to explore the support for diagnosis nature of the molecular markers under study [19]. Binary and multinomial logistic regression analysis was used to investigate the influence of molecular markers on the clinical variables. All the clinical variables were converted to categorical variables in the whole regression analysis. Cox proportional hazards regression analysis was undertaken in case of continuous variables, such as *COL19A1* levels, to evaluate their prognostic nature. Patients who were alive at the time of analysis were censored. After identifying significant predictors of prognosis on multivariate analysis, internal validation of the model was carried out using boot-strapping techniques using 1000 random samples to obtain 95 % confidence. Leave-one-out cross validation was used to confirm the accuracy in each regression analysis. Linear mixed-effects models were used to estimate the influence of *COL19A1* levels on outcomes in the longitudinal study. The fixed effect of the baseline value of the outcome and the period of time in months on outcomes was modeled. Random intercepts were used to model between-patients variability. Statistical analysis was performed using SPSS Statistics version 22 (IBM, Spain). A two-sided significance level of 5% was considered statistically significant.

## RESULTS

### COL19A1 levels were strongest predictors of survival in the muscle biopsy cohort

**Figure 2.**
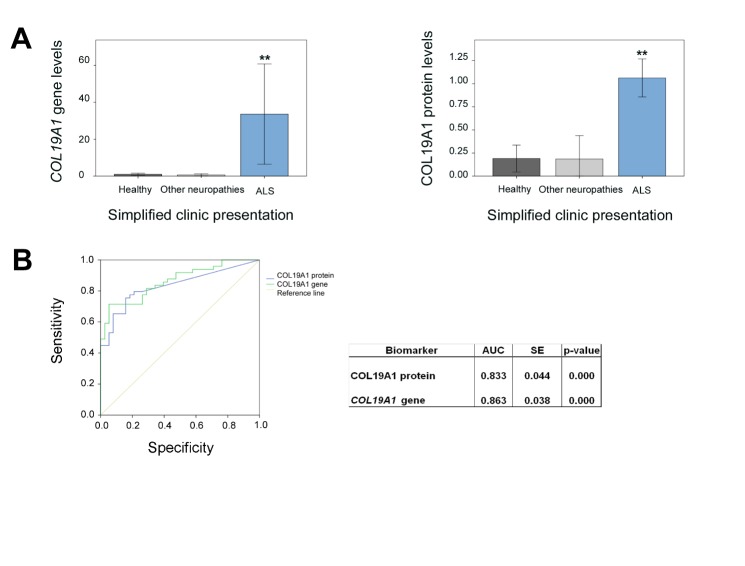
Relative gene and protein expression levels of COL19A1 in the muscle biopsy study cohort. Gene and protein expression levels of COL19A1 in healthy subjects, ALS patients and other neuropathies patients (A). Kruskal Wallis tests showed significant differences among ALS patients and the other two groups under study, when gene and protein expression levels were tested (P < 0.001, **). (B) Areas under ROC curves (AUC) of gene and protein expression of COL19A1 were calculated to test its support for diagnosis criterion in ALS patients (P <0.001, **). A total of 49 ALS (FALS and SALS) participants were included in this study and matched with 24 control individuals, and 14 ONP; SE: standard error.

The expression profile of fifteen genes and fourteen proteins was analyzed in the muscle biopsy cohort and compared to healthy subjects and to ONP groups. Only *COL19A1* gene and protein levels were significantly different in the ALS patient group with respect to healthy controls and the ONP groups. Remarkably, COL19A1 protein levels were approximately five-fold increase in the ALS patient group respect to healthy controls and the ONP groups in, while *COL19A1* gene levels were approximately thirty-fold increase in ALS patients respect to the control groups (Fig. 2A). Furthermore, comparison of *COL19A1* expression profile with the Area under ROC curves (AUC) was performed to test if *COL19A1* levels could have a good performance as support for diagnosis tool. In order to assess predictive values, selected cut-off values were set for *COL19A1* gene (*COL19A1* gene >7), and for COL19A1 protein (COL19A1 protein >1) to minimize false positive cases (Fig. 2B). The parallel combination of data improved performance related to predictive values, yielding a 71.0% sensitivity and 100% specificity with a positive predictive value (PPV) of 99.9% and a negative predictive value (NPV) of 99.9% (likelihood ratio, LR: 6.00, confidence interval, CI: 2.471-14.571).

We next explored the prognostic nature of *COL19A1* gene in relation to the clinical parameters measured in ALS patients. The ALSFRS-r score provides an estimation of the patient’s degree of functional impairment. Since the 1990s, this functional rating scale has been developed to measure the disease progression in the patients from time at onset to time at diagnosis and follow-up visits in the clinic. This scale is based on a set of twelve questions graded on a scale of 0 to 4. The responses obtained in this questionnaire can shed light on the different domains that might be affected by the disease. The new and revised scale considers 48 points as the maximum score for each question [20]. It has been described that the rate of deterioration in the patient correlates with survival and, at the same time, it also correlates with a lower score in the ALSFRS-r scale, suggesting approximately an average loss of a point per month along the disease progression and therefore a higher disability in the patient [20]. Spearman correlation between ALSFRS-r score at sampling and *COL19A1* gene levels was found significant (r= -0.349, *P* =0.025), which suggested that high *COL19A1* levels could be related to lower ALSFRS-r scores at sampling and a higher disability in the patient. Binary logistic regression analysis showed that ALS patients that exhibited ALSFRS-r scores at sampling higher or equal than 40, also showed a probability of expressing lower *COL19A1* gene and protein levels than ALS patients with ALSFRS-r scores at sampling lower than 40 (*COL19A1* gene levels: odds ratio (OR): 0.839, 95% confidence of interval (CI): 0.714-0.987, *P* =0.034; COL19A1 protein levels: OR: 0.977, CI: 0.877-1.089, *P* =0.674). In addition, Spearman correlation between disease duration and diagnostic delay (time from symptom onset to diagnosis) was found significant (r= 0.579, *P* <0.0001) as well as between disease duration and ALSFRS-r slope at sampling (r= -0.761, *P* <0.0001) and between diagnostic delay and ALSFRS-r slope at sampling (r= -0.556, *P* <0.0001). In this sense, ALS patients that showed a longer diagnostic delay (more than one year) also showed longer disease duration and a slow progression of the disease (lower ALSFRS-r slope scores, lower than 1 points/month).

**Figure 3.**
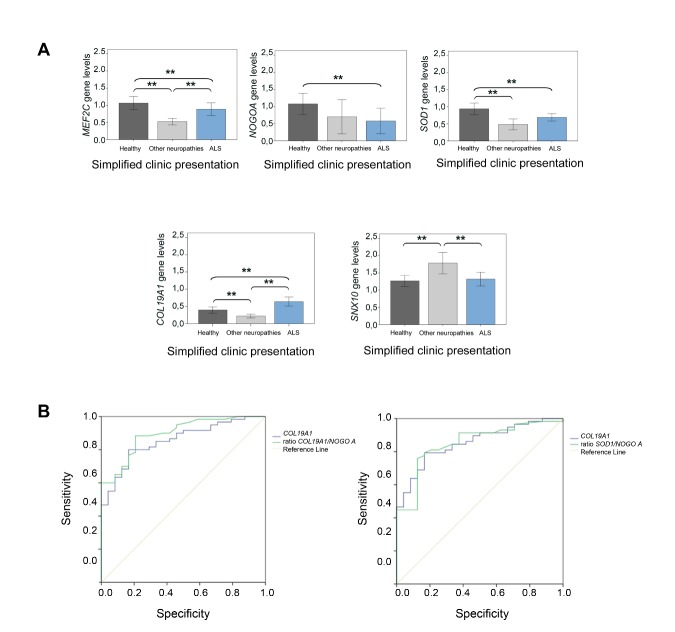
Relative gene expression analysis in the blood study cohort. Transcriptional expression levels of *MEF2C* , *NOGO A* , *SOD1* , *COL19A1* and *SNX10* in healthy subjects, ALS patients and other neuropathies patients. (A) Kruskal Wallis tests showed significant differences among ALS patients and healthy participants in all the cases except for *SNX10* . Significant differences among other neuropathies patient group and ALS patient group were found in all the cases except for *NOGO A* and *SOD1* . (B) Area under ROC curves of *COL19A1* and the ratios *COL19A1* /*NOGO A* and *SOD1* /*NOGO A* were calculated in the sporadic ALS patient group. Significant differences were found between *COL19A1* and the ratio *COL19A1* /*NOGO A* , and between *COL19A1* and the ratio *SOD1* /*NOGO A* . A total of 141 participants were included in this study: 58 control individuals, 24 other neuropathology’s individuals and 59 ALS patients (FALS and SALS patients); (*P < 0.05; ** P < 0.001), SE: standard error.

The next step was to investigate the role of *COL19A1* levels in the disease progression, the diagnostic delay and the ALSFRS-r slope at sampling. Linear regression analysis suggested the disease duration correlated positively with the diagnostic delay (B coefficient: 0.820, CI: 0.452-1.189, *P* =0.008) and negatively with the ALSFRS-r slope (B coefficient: -10.587, CI: -19.586-(-1.588), *P* =0.023) and with low COL19A1 protein levels (B coefficient: -14.407, CI: -30.228-1.414, *P* =0.043), suggesting the higher ALSFRS-r slope scores (faster progression of the disease) and the higher COL19A1 protein levels, the shorter disease duration. Additionally, the diagnostic delay correlated positively with the disease duration in ALS patients that exhibited low *COL19A1* gene levels (B coefficient: 0.909, CI: 0.556-1.263, *P* <0.0001), suggesting that low *COL19A1* gene levels were closely associated with ALS patients that showed a longer disease duration and therefore, a diagnostic delay higher than one year.

Finally, to validate the prognostic nature of COL19A1 expression levels multivariable Cox proportional hazards regression model was performed using leave-one-out cross validation. This analysis suggested that the lifespan in ALS patients showing COL19A1 protein levels above mean average (1.06) and additionally a fast progression of the disease (ALSFRS-r slope scores at sampling ≥ 1 points/month) could be reduced 70.5% (ALSFRS-r slope, hazard ratio, HR: 1.569, CI: 1.245-1.976, *P* =0.000; COL19A1 protein levels, HR: 2.158, CI: 1.020-4.566, *P* =0.044), emphasizing the significance of *COL19A1* levels as a useful, feasible, and potentially prognostic factor in patients with ALS. Since *COL19A1* levels were found strongest predictors of survival in the ALS muscle biopsy cohort, we next investigated a second cohort of patients, the blood cohort, to validate these findings.

**Figure 4.**
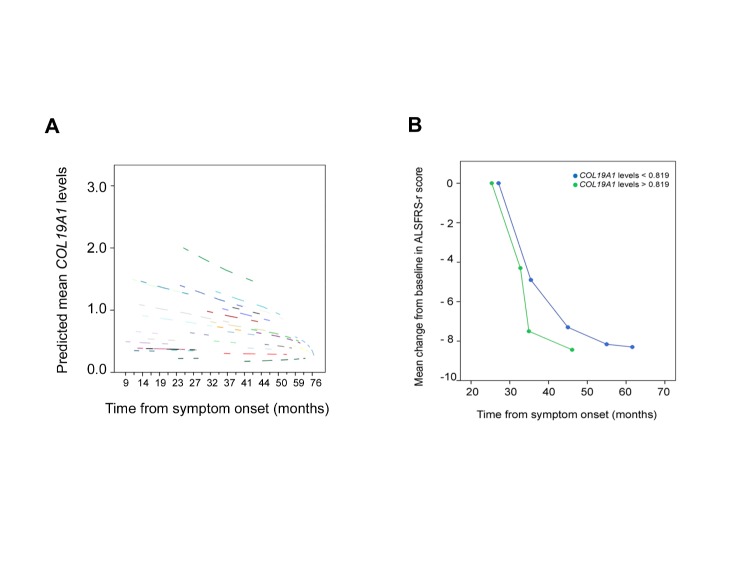
Linear mixed model analysis in the serial blood study cohort. (A) Linear mixed model analysis showed a significant relationship among a faster disease progression and high *COL19A1* levels from symptom onset, each dashed line corresponds to the *COL19A1* levels from each patient. (B) Mean change from baseline in the ALSFRS-r score in patients that were monitored at 6-monthly intervals during a follow-up period of 24 months from the symptom onset that show *COL19A1* levels above (green dot and line) and below (blue dot and line) average at first evaluation. Student's-t test was performed to analyze statistical differences between groups (P < 0.001). A total of 40 SALS patients from the serial blood study cohort were included.

### The prognostic nature of COL19A1 levels was also validated in blood samples from ALS patients

The gene expression profile of fifteen genes was analyzed in the ALS blood cohort and compared to the healthy group of subjects and ONP patients. Results showed that only *COL19A1* and *MEF2C* levels were significantly deregulated in the ALS patient group compared to healthy controls and the ONP groups (Fig. 3A). Albeit *COL19A1* levels were found significantly higher in the ALS patient group respect to the healthy and ONP groups, this difference in the gene expression levels was not so remarkable than the difference observed in the muscle biopsy cohort (approximately 1.75-fold change respect to the healthy control group and 2.35-fold change respect to the ONP group), which is in accordance with the fact that *COL19A1* gene is mostly expressed and related to the skeletal muscle tissue, rather than blood tissue. ROC curves analysis showed that the sensitivity and specificity ratios between *COL19A1* and *COL19A1* /*NOGO A* and *SOD1* /*NOGOA* ratios were found statistically significant in the sporadic ALS patients’ group (Fig. 3B). Selected cut-off values were set for *COL19A1* gene (*COL19A1* gene >0.847), and for *COL19A1* /*NOGO A* ratio (ratio *COL19A1* /*NOGO A* >0.650), as well as for *SOD1* /*NOGO A* (ratio *SOD1* /*NOGO A* >0.960) to minimize false positive cases. These results showed that a parallel combination of *COL19A1* and the ratio *COL19A1/NOGO A* improved performance, yielding a 78.7% sensitivity, while in the case of *COL19A1* and the ratio *SOD1/NOGO A* , the sensitivity reached 70.7% (LR 6.214, CI 2.449-15.771). Consequently, the relationship between *COL19A1* levels and the ratios *COL19A1* /*NOGO A* and *SOD1* /*NOGO A* could be more indicative of muscle damage in the population of sporadic ALS patients.

Our next step was to investigate the relationship of these genes with the functional state in ALS patients. Spearman correlations between ALSFRS-r score at onset and *COL19A1* levels (r= -0.345, *P* =0.001), *MEF2C* (r= 0.228, *P* =0.038) and *SNX10* (r= 0.494, *P* =0.000) gene levels were found significant, which suggested that high *COL19A1* levels could be related to lower ALSFRS-r scores, indicating a high disability in the patient, while high *MEF2C* and *SNX10* levels were more associated with a lower disability in the patient. Furthermore, *COL19A1* levels correlated negatively with the diagnostic delay (r= -0.210, *P* =0.040), reinforcing that *COL19A1* levels could be involved in a faster progression of the disease.

To investigate more in depth these correlations, binary logistic regression analysis was performed. We next explored the possible associations of these genes and the clinical variables measured in each patient that included ALSFRS-r at onset and at sampling, the diagnostic delay, age at onset, the revised El Escorial criteria, the onset site and the genetic diagnosis.

*COL19A1* levels were associated with an age at onset lower than 55 years (OR: 2.967, CI: 1.213-7.257, *P* =0.017), a defined El Escorial criteria (OR: 3.050, CI: 1.266-7.350, *P* =0.013) and ALSFRS-r scores equal or lower than 40 (OR: 2.433, CI: 0.970-6.105, *P* =0.058), which is in accordance with the results observed in the muscle biopsy cohort. Therefore, high *COL19A1* levels in blood could be also indicative of a worsening of the functional state and a high disability in the patient. Additionally, *COL19A1* levels were also associated with a diagnostic delay lower than one year (OR: 0.953, CI: 0.909-0.998, *P* =0.043). In contrast, *SNX10* and *NOGO A* levels were more associated with ALSFRS-r scores at onset higher than 40 (*SNX10* , OR: 0.381, CI: 0.213-0.679, *P* =0.001; *NOGO A* , OR: 0.366, CI: 0.184-0.725, *P* =0.004), and an age at onset higher than 55 years (OR: 0.333, CI: 0.167-0.665, *P* =0.002), suggesting that high *SNX10* and *NOGO A* levels could be related to a lower disability in the patient. Interestingly, only in the case of *MEF2C* levels, a significant association was found between high levels of this gene and the bulbar and upper limb onset site of the disease (OR: 2.963, CI: 1.171-7.497, *P* =0.022), which could indicate an advanced muscle damage. In addition, *COL19A1* levels were associated with the sporadic form of the disease (OR: 13.771, CI: 2.520-75.255, *P* =0.002), while *SNX10* levels were more associated with the familial form of the disease (OR: 0.454, CI: 0.268-0.767, *P* =0.003).

Our next step was to unravel which of these genes could influence the clinical features in the patients and therefore, multivariate logistic regressions were performed. *SNX10* levels were found significantly associated with the familial ALS patients’ group (OR: 0.489, CI: 0.268-0.892, *P* =0.020), with ALS patients with an age at onset higher than 55 years (OR: 0.351, CI: 0.173-0.714, *P* =0.004), and with patients showing a possible, probable or suspected El Escorial criteria at onset (OR: 0.417, CI: 0.211-0.822, *P* =0.012). These findings suggested that SNX10 levels could be indicative of a late worsening in the functional state of the ALS patients. In contrast, *COL19A1* levels were found significantly associated with sporadic ALS patients (OR: 16.106, CI: 2.294-113.053, *P* =0.005), with patients that share an age at onset lower than 55 years (OR: 2.543, CI: 1.007-6.422, *P* =0.048), and a defined El Escorial criteria (OR: 2.955, CI: 1.214-7.192, *P* =0.017), highlighting that *COL19A1* levels could be more indicative of an earlier worsening in the functional state in the ALS patients.

Finally and after analyzing the prognostic nature of the genes along the disease progression, Cox proportional hazards regression model suggested that *COL19A1* gene levels above average (0.819) and a fast progression of the disease, based on high scores of the ALSFRS-r slope at onset (≥ 1 points/month) could favored a shorter lifespan in ALS patients (ALSFRS-r slope, HR: 5.798, CI: 3.405-9.872, *P* <0.001; *COL19A1* levels, HR: 2.291, CI: 1.435-5.945, *P* =0.003). Notwithstanding, ALSFRS-r slope at sampling was also considering to be a potential predictor of disease progression, improving the accuracy of the prognostic prediction. Cox regression analysis, using leave-one-out cross validation, suggested that *COL19A1* gene levels above mean average (0.819) and high ALSFRS-r slope at sampling scores above mean average (≥ 1 points/month) could promote a 20% reduction in the lifespan of ALS patients (*COL19A1* , HR: 2.114, CI: 1.678-2.772, *P* =0.019, ALSFRS-r slope at sampling, HR: 2.368, CI: 1.763-3.181, *P* <0.001).

These findings reinforced the potential role of *COL19A1* levels as strongest predictors of disease progression in this cross-sectional study, in which only one sample of blood was extracted from each participant and analyzed. The validation of these findings in serial blood samples obtained in a longitudinal study could finally unravel the prognostic nature of *COL19A1* levels in ALS patients, and especially in sporadic cases to which *COL19A1* was significantly associated.

### High COL19A1 levels enabled a more precise prognosis in patients that shared a high disability in the first symptomatic stages

Blood serial samples obtained from the same patient can provide useful information about the variation of the expression profile of any molecular marker along the time. In addition, they can be easily monitored and studied in parallel with the clinical variables that each patient exhibit during the progression of the disease, especially the ASFRS-r scores and ALSFRS-r slope, in a longitudinal study. Our hypothesis at this step was based on validating the prognostic nature of *COL19A1* levels in blood serial samples along the disease progression. For this purpose, both time-dependent Cox regression analysis and linear mixed models were performed in the serial blood cohort. The linear mixed model analysis showed that ALS patients that exhibited high *COL19A1* levels from symptom onset also progressed faster during the follow-up period of 24 months from symptom onset (Estimate: -0,0093, CI: (-0,0181) - (-0,00039); *P* =0,042) (Fig. 4A). In addition, time-dependent Cox regression analysis, using leave-one-out cross validation, suggested that the lifespan of sporadic ALS patients showing *COL19A1* levels above average (0.819) and a faster progression of the disease (ALSFRS-r slope at sampling scores ≥ 1 points/month) was reduced 17.9% (HR: 1.179, CI: 1.046-1.327, *P* =0.007), similarly to the result obtained in the blood cohort. Interestingly, ALS patients that showed *COL19A1* levels below average from symptom onset (0.819) also showed a longer life expectancy and a light and progressive change in the ALSFRS-r scores at sampling in the follow-up period along disease progression than patients that showed *COL19A1* levels above average (Fig. 4B). In accordance with the results obtained in the muscle biopsy and blood cohorts, these findings highlighted the prognostic nature of *COL19A1* levels in ALS patients that exhibited both *COL19A1* levels above average and ALSFRS-r slope at sampling scores ≥ 1 points/month in the serial blood cohort, suggesting that *COL19A1* levels could be of valuable support in clinical practice when ALSFRS-r slope at sampling was also considered to monitor the ALS patients’ group.

The prognostic nature of *COL19A1* levels in the three cohorts under study is summarized in terms of the average values in *COL19A1* levels and the corresponding ALSFRS-r slope at sampling scores (Table 6).

**Table 6:** Prognostic nature of *COL19A1* levels in the three cohorts under study.

Group of ALS patients	N	*COL19A1* levels	ALSFRS-r slope	Estimate	HR (95% CI)	P value
Muscle cohort*	49	above the mean average (1.06)	≥ 1 points/month	0.203	1.225 (1.058 - 1.418)	0.007
Blood cohort	59	above the mean average (0.819)	≥ 1 points/month	0.748	2.114 (1.134 - 3.941)	0.019
Serial blood cohort	40	above the mean average (0.819)	≥ 1 points/month	0.164	1.179 (1.046 - 1.327)	0.007

Cox regression analysis, using leave-one-out cross validation, suggested that in the group of ALS patients, both *COL19A1* levels above the mean average at first evaluation and ALSFRS-r slope at sampling scores ≥ 1 points/month in each corresponding cohort of patients were associated with a shorter lifespan. N: number of enrolled ALS patients; ALSFRS-r slope at sampling; HR: hazard ratio; CI: confidence interval; * only in the case of the Muscle biopsy cohort, the COL19A1 levels corresponds to the protein levels. In the Blood cohort the finding was reproducible when using the average score for *COL19A1* gene levels of 0.819, used in the Serial Blood cohort (P < 0.05, *; P < 0.001, **).

### DISCUSSION

This study was designed and performed to identify more reliable prognostic biomarkers for ALS disease progression that could be in close connection with the clinical parameters for a better monitoring and stratification of the patients. The starting point in this study was the skeletal muscle tissue although we also explored blood tissue to identify and validate prognostic biomarkers in a non-invasive way.

In muscle biopsy samples, *COL19A1* gene and protein levels were significantly associated with the ALS patient group with respect to healthy controls and patients with ONP, suggesting that *COL19A1* levels can be useful as molecular support for diagnosis in this tissue. In the ALS group, high *COL19A1* gene levels were significantly associated with low ALSFRS-r scores, and high COL19A1 protein levels promoted a shorter lifespan in ALS patients that also showed high ALSFRS-r slope scores, and consequently, a faster disease progression. A similar situation was observed in blood samples. *COL19A1* and *MEF2C* levels were associated with the ALS patient group with respect to healthy controls and patients with ONP. In the ALS group, high *COL19A1* levels in combination with age at onset, the revised El Escorial criteria, the diagnostic delay and ALSFRS-r scores were associated with a worse prognosis indicating a high disability in the patient. In contrast, high *NOGO A* and, especially *SNX10* levels were related to a better prognosis in ALS patients. However, the prognostic nature of these potential biomarkers in blood serial samples were only validated in the case of *COL19A1* levels since patients that exhibited high *COL19A1* levels and a faster disease progression also showed a shorter lifespan.

Collagen XIX accumulates in the extracellular matrix of restricted tissues, including neural tissue and it seems to modulate cell-matrix interactions and cell-cell communications [14,21]. Recently, the anti-tumor properties of *COL19A1 in vitro* have also been described, albeit it is involved in the differentiation of muscle cells, central nervous system development, and formation of the esophagus [22]. When muscle fibers are functionally denervated, as it happens in ALS disease, the subsynaptic membrane is able to restore its biochemical and structural organization [23]. The findings observed in muscle biopsy samples suggested that ALS patients that exhibited a faster disease progression they could also exhibited more severe muscle damage. Therefore, high levels of *COL19A1* could act as a compensatory response when the disease progression is fast, which is in accordance with previous studies that suggested a close relationship between *COL19A1* gene and ALS in muscle biopsies from ALS patients [24]. Furthermore, the remarkable difference in *COL19A1* gene and protein levels observed in the ALS patients respect to the ONP group also suggested that *COL19A1* could be involved in specific muscle defects that could enhance the desestabilization of motor neuron terminals, contributing especially to the neurodegenerative progression in ALS. Albeit the neuropathies included in this study also share in common with ALS intrinsic muscle defects, neuroinflammation, immune organ dysfunction, metabolic perturbations, defects in neuron excitability and selective motoneuron vulnerability and muscle denervation process, specific processes such as activation of regulatory myogenic factors [14] or the direct cross-talk between NMJ and axonal outgrowth could not be modulated in the same way as in ALS. Structural or functional alterations of skeletal muscle can hinder the transmission of chemical and electrical stimuli from the NMJ to the motor neurons, favoring the dying-back phenomena and therefore contributing to disease pathology [25]. Since COL19A1 is a matrix protein involved in muscle physiology and differentiation [14], alterations in the *COL19A1* gene or protein levels could point out tissue damage along disease progression in the neurodegenerative chain of ALS.

In blood samples, high levels of *COL19A1* could also represent a repressor stimulus to counteract the denervation processes along disease progression [9,23], and even an overstimulation of immune system in monocytes and dendritic cells by means of leukocyte-associated immunoglobulin-like receptor 1 (LAIR-1) [26]. Recent studies have suggested a novel function of *SNX10* in regulating macrophage polarization and function in mice under inflammatory conditions [27]. Considering that *SNX10* has also been reported to be associated with a diminished production of systemic pro-inflammatory cytokines [27], increasing *SNX10* levels could favor macrophage polarization into M2 phenotype, exerting anti-inflammatory action after tissue injury in ALS patients, when the denervation process has started as increasing *NOGO A* levels suggested, which is in accordance with previous studies [28-32]. Remarkably, it has been reported that thirteen circulating markers of inflammation and of neuromuscular pathology changes were significantly altered in plasma from ALS patients, highlighting the role of inflammatory response and neuromuscular involvement in ALS [33,34]. Moreover, this is an interesting issue since only one recent study has identified blood-derived biomarker from peripheral lymphocytes in ALS patients. In this study, *LILRA2* , *ITGB2* and *CEBPD* genes were identified as peripherally accessible candidate biomarkers in ALS [35]. These genes are related to the immune system but also they are in close connection with the crosstalk between microglia and motor neuron pathology. The clinical relevance of identifying blood-derived biomarkers from peripheral lymphocytes mainly relies on the fact that it is possible to monitor the impact of other tissues and systems that become damaged during disease progression, such as neuroinflammation, microglial activation or even muscle damage, in parallel with the clinical parameters that can be serially monitored in the patients, at the same time the expression profile of the biomarker candidate is tested in a non-invasive way. In this study, *COL19A1* gene improved prognosis accuracy during disease progression not only in patients that were monitored at first evaluation in the blood cohort but also in serial samples and in patients that were monitored longitudinally at 6-monthly intervals during a follow-up period of 24 months, highlighting the prognostic role of this gene in the disease progression and in particular, in the sporadic form of the disease.

The findings provided in this study revealed the potential use of *COL19A1* to improve prognosis in the disease progression in a non-invasive way. The combination of high *COL19A1* expression levels and a faster disease progression can promote a shorter life expectancy in ALS patients, and therefore *COL19A1* levels can be considered a reliable blood-derived biomarker in muscle biopsies and in blood to support the clinical practice and to be of help in future clinical trials, as well as a promising and novel therapeutic target in ALS.
